# Commentary: The contribution of personalized video feedback to robotic partial nephrectomy training in realistic 3D tumor kidney models: design, production and implementation

**DOI:** 10.3389/fsurg.2025.1738938

**Published:** 2026-01-02

**Authors:** Wenjiang Yang

**Affiliations:** Department of Urological Surgical, Wenshang County People’s Hospital, Jining, Shandong, China

**Keywords:** robotic partial nephrectomy, 3D-Printed kidney models, personalized video feedback, surgical simulation, surgical education

A Commentary on The contribution of personalized video feedback to robotic partial nephrectomy training in realistic 3D tumor kidney models: design, production and implementation By Sarıkaya AF, Tarım K, Köseoğlu E, Özkan A, Aykanat İC, Esen B, et al. (2025). Front Surg. 12:1615817. doi: 10.3389/fsurg.2025.1615817

I read with great interest the manuscript by Sankaya et al., published in Frontiers in Surgery, which investigates the innovative integration of low-cost, realistic 3D-printed kidney models and personalized video feedback for robotic partial nephrectomy (RAPN) training (1). The authors present a compelling study demonstrating that this combined approach significantly improves surgical precision and dissection skills among urology residents. The focus on cost-effectiveness, standardized model production, and objective skill assessment is a noteworthy contribution to the field of simulation-based surgical education, aligning with the growing emphasis on proficiency-based progression (2).

The study's findings are encouraging. The demonstration that personalized video feedback led to a statistically significant improvement in the percentage reduction of dissection time (46.63% vs. 23.62%, *p* = 0.043) and a significant decrease in the amount of healthy parenchyma removed (*p* = 0.048) provides strong evidence for the efficacy of this training modality. Furthermore, the authors' successful development of a highly cost-effective model ($2.14 per unit) addresses a critical barrier to the widespread adoption of high-fidelity simulation, making such training more accessible and scalable (3).

However, while the study is robust in its design and execution, several aspects warrant further discussion to fully contextualize the findings and their generalizability:

## Long-term skill retention and transferability

The study effectively demonstrates short-term skill improvement between two consecutive procedures on identical models. A key question that remains is the durability of this acquired skill. As highlighted in systematic reviews, the ultimate validation for any simulation training is the transfer of skills to the operating room and the demonstration of long-term retention (4). Future studies with longitudinal follow-up and assessment of intraoperative performance would be invaluable in confirming the sustained clinical impact of this training method.

## Model fidelity and unsimulated challenges

The authors rightly acknowledge the limitation of being unable to simulate intraoperative bleeding. This is a critical shortcoming in the context of RAPN, where effective hemostasis and subsequent renorrhaphy are central to preventing complications and minimizing warm ischemia time—key components of the “Trifecta” outcomes (Warm Ischemia Time, Estimated Blood Loss, and Negative Surgical Margins). The current model, while excellent for teaching dissection planes, does not adequately prepare trainees for the hemostatic and reconstructive phases of the procedure, which are essential for achieving true procedural proficiency. As seen in other high-fidelity simulations, incorporating perfused features or simulated bleeding can significantly enhance the realism and training value for complex procedures (5). The absence of these elements limits the model's ability to fully replicate the intraoperative environment and thus constrains the generalizability of the findings to actual clinical performance. The addition of such elements in future iterations could address this gap and provide a more holistic training platform. This aligns with the findings of Antonio AG et al. (2024), who recently emphasized that while 3D-printed models excel in anatomical representation and preoperative planning, their utility for comprehensive skill assessment remains limited without the integration of critical functional elements like perfusion and realistic tissue behavior (6).

## Standardization and scalability of feedback

The video feedback was provided by a single, experienced robotic surgeon to ensure consistency. For this methodology to be scalable, a more standardized framework for feedback would be beneficial. The development of structured, objective assessment tools, similar to those used in other robotic surgery metrics initiatives, could help reduce inter-instructor variability and facilitate wider implementation (7).

In conclusion, the work by Sankaya and colleagues represents a significant step forward in optimizing surgical simulation. Their model successfully combines technological innovation with a powerful educational principle. The results convincingly show that this approach can enhance technical proficiency. However, the limitations in model fidelity—particularly the lack of bleeding simulation—and the short-term nature of the skill assessment must be addressed in future studies to validate the clinical relevance and transferability of this training paradigm.

## References

[1] Sarıkaya AF Tarım K Köseoğlu E Özkan A Aykanat İC Esen B The contribution of personalized video feedback to robotic partial nephrectomy training in realistic 3D tumor kidney models: design, production and implementation. Front Surg. (2025) 12:1615817. 10.3389/fsurg.2025.1615817 40746836 PMC12310688

[2] Gallagher AG De Groote R Pacioni M Mottrie A. Proficiency-based progression training: a scientific approach to learning surgical skills. Eur Urol. (2022) 81(4):394–5. 10.1016/j.eururo.2022.01.004 35074249

[3] Jiang Y Jiang H Yang Z Li Y. The current application of 3D printing simulator in surgical training. Front Med (Lausanne). (2024) 11:1443024. 10.3389/fmed.2024.1443024 39267979 PMC11390463

[4] Mazzone E Puliatti S Amato M Bunting B Rocco B Montorsi F A systematic review and meta-analysis on the impact of proficiency-based progression simulation training on performance outcomes. Ann Surg. (2021) 274(2):281–9. 10.1097/SLA.0000000000004650 33630473

[5] Ghazi A Melnyk R Farooq S Bell A Holler T Saba P Validity of a patient-specific percutaneous nephrolithotomy (PCNL) simulated surgical rehearsal platform: impact on patient and surgical outcomes. World J Urol. (2022) 40(3):627–37. 10.1007/s00345-021-03766-7 34165633 PMC9796494

[6] Grosso AA Di Maida F Lambertini L Cadenar A Coco S Ciaralli E Three-dimensional virtual model for robot-assisted partial nephrectomy: a propensity-score matching analysis with a contemporary control group. World J Urol. (2024) 42(1):338. 10.1007/s00345-024-05043-9 38767673 PMC11106151

[7] Mottrie A Mazzone E Wiklund P Graefen M Collins JW De Groote R Objective assessment of intraoperative skills for robot-assisted radical prostatectomy (RARP): results from the ERUS scientific and educational working groups metrics initiative. BJU Int. (2021) 128(1):103–11. 10.1111/bju.15311 33251703 PMC8359192

